# The Subthalamic Microlesion Story in Parkinson's Disease: Electrode Insertion-Related Motor Improvement with Relative Cortico-Subcortical Hypoactivation in fMRI

**DOI:** 10.1371/journal.pone.0049056

**Published:** 2012-11-07

**Authors:** Robert Jech, Karsten Mueller, Dušan Urgošík, Tomáš Sieger, Štefan Holiga, Filip Růžička, Petr Dušek, Petra Havránková, Josef Vymazal, Evžen Růžička

**Affiliations:** 1 Dept. of Neurology and Center of Clinical Neuroscience, Charles University in Prague, 1st Faculty of Medicine and General University Hospital, Prague, Czech Republic; 2 Max Planck Institute for Human Cognitive and Brain Sciences, Leipzig, Germany; 3 Dept. of Stereotactic and Radiation Neurosurgery, Na Homolce Hospital, Prague, Czech Republic; 4 Department of Cybernetics, Czech Technical University in Prague, Faculty of Electrical Engineering, Prague, Czech Republic; 5 Department of Radiology, Na Homolce Hospital, Prague, Czech Republic; Beijing Institute of Radiation Medicine, China

## Abstract

Electrode implantation into the subthalamic nucleus for deep brain stimulation in Parkinson's disease (PD) is associated with a temporary motor improvement occurring prior to neurostimulation. We studied this phenomenon by functional magnetic resonance imaging (fMRI) when considering the Unified Parkinson's Disease Rating Scale (UPDRS-III) and collateral oedema. Twelve patients with PD (age 55.9± (SD)6.8 years, PD duration 9–15 years) underwent bilateral electrode implantation into the subthalamic nucleus. The fMRI was carried out after an overnight withdrawal of levodopa (OFF condition): (i) before and (ii) within three days after surgery in absence of neurostimulation. The motor task involved visually triggered finger tapping. The OFF/UPDRS-III score dropped from 33.8±8.7 before to 23.3±4.8 after the surgery (*p*<0.001), correlating with the postoperative oedema score (*p*<0.05). During the motor task, bilateral activation of the thalamus and basal ganglia, motor cortex and insula were preoperatively higher than after surgery (*p*<0.001). The results became more enhanced after compensation for the oedema and UPDRS-III scores. In addition, the rigidity and axial symptoms score correlated inversely with activation of the putamen and globus pallidus (*p*<0.0001). One month later, the OFF/UPDRS-III score had returned to the preoperative level (35.8±7.0, *p* = 0.4).

In conclusion, motor improvement induced by insertion of an inactive electrode into the subthalamic nucleus caused an acute microlesion which was at least partially related to the collateral oedema and associated with extensive impact on the motor network. This was postoperatively manifested as lowered movement-related activation at the cortical and subcortical levels and differed from the known effects of neurostimulation or levodopa. The motor system finally adapted to the microlesion within one month as suggested by loss of motor improvement and good efficacy of deep brain stimulation.

## Introduction

Deep brain stimulation of the subthalamic nucleus (STN DBS) has become an effective treatment for Parkinson's disease (PD) [1]. According to previous reports, insertion of the intracerebral electrode into the brain tissue itself may contribute to clinical improvement from DBS [2], [3], [4]. The lesion can even be used for therapeutic purposes as documented by clinical effects of conventional thermo-coagulation subthalamotomy [5], [6]. On the contrary, a micro-subthalamotomy from DBS electrode implantation is manifested by smaller, though still demonstrable motor improvement in the absence of neurostimulation. It is usually already manifested during the surgery by a mild decrease of rigidity, akinesia and tremor and can remain noticeable for months after implantation in the OFF state [7]. Alleviation of off-dystonia [8] and worsening in verbal fluency [9] are other possible consequences. The size of microlesion depends on the surgical techniques as the size of the microlesion grows parallel to the number of micro-electrodes passing through STN in correlation to postoperative motor improvement [10].

The micro-subthalamotomy effect induced by DBS electrode insertion was previously studied by positron emission tomography (PET) showing altered glucose metabolism detectable even one year after implantation. Under resting state, with dopaminergic drugs withdrawn and stimulation switched off, there was a deactivation of the subthalamus, [11] thalamus and basal ganglia together with hyperactivation of the sensorimotor cortex and cerebellum, [12] thus making the pattern similar to conventional subthalamotomy [13], [14]. Up to now, none of the functional imaging studies evaluated the STN microlesion effects during voluntary movements, despite similarly oriented papers on the effects of DBS or levodopa [15], [16], [17], [18].

We used a functional magnetic resonance imaging (fMRI) based on the blood oxygenation level-dependent (BOLD) signal acquired before and shortly after the implantation of cerebral electrodes into the STN. Admittedly, MRI examination poses the risk of thermal brain damage from a radiofrequency field arising from the presence of the metallic implant [19], [20]. On the other hand, an MRI is often used by many implantation teams for confirming the position of implanted electrodes, or for structural brain imaging in cases of postoperative complications. Nevertheless, its use in hundreds of implanted patients [21], [22], [23] may serve as evidence that with adherence to defined precautions [24], [25] an MRI can be considered safe even in the presence of an intracerebral electrode [26]. The same applies to fMRI which can be used for detection of local as well as distant effects of DBS on the cerebral cortex [27]. We were the first to introduce fMRI into the study of STN DBS effects in PD, [28] a step successfully followed by others [29], [30], [31], [32].

In the present study, we searched for any differences in motor system activation patterns caused by bilateral insertion of electrodes into the STN in the absence of medication or neurostimulation. Unlike previous studies, we explored the microlesion phenomenon while using an active motor task involving finger tapping well known from the Unified Parkinson's Disease Rating Scale (UPDRS-III). We hypothesized that apart from neuronal loss caused by electrode insertion, clinical consequences would also arise from a collateral oedema developing around the electrode [3], [33], [34]. Therefore, we introduced simple scales for quantifying the extent of cortical and subcortical oedema to compare it with the postoperative changes in the UPDRS-III and fMRI. Finally, we compared the clinical symptoms in STN DBS ON condition with preoperative effects of levodopa and analyzed the local subthalamic lesion volume with regard to the number of microelectrodes hitting the STN and to the number of macroelectrode insertions used for clinical verification of DBS during surgery.

## Methods

### Subjects

Twelve PD patients were enrolled consecutively in 2009–2010. All of them were suffering from motor fluctuations and/or disabling dyskinesias (demographic details in Table 1 and Table S1) and were indicated for treatment with STN DBS. All of them met the UK Brain Bank Criteria for diagnosis of PD and all gave their written informed consent for participation. Patients with dementia and/or depression had been excluded by routine psychiatric examination and neuropsychological testing (Mini-mental state examination, Mattis dementia rating scale, Beck depression inventory). The study was approved by the Ethics Committee of the General University Hospital in Prague, Czech Republic.

**Table 1:** Description of the PD patient's group (N = 12).

age (years):	55.9±6.8	45–64
gender M/F:	12/0	
duration of the PD (years):	12.4±2.0	9–15
duration of levodopa treatment (years):	9.3±2.7	5–13
duration of motor complications (years):	5.0±2.9	2–12
MMSE:	28.9±1.0	28–30
**UPDRS-III**		
UPDRS-III – before implantation (session 1)		
OFF state:	33.8±8.7	
mON state:	9.8±4.5	
UPDRS-III – within 1–3 days after implantation (session 2)		
OFF state:	23.3±4.8	
sON state:	9.3±2.6	
DBS parameters:	2.8±0.3 V	60 µs, 130 Hz, bipolar mode
UPDRS-III – one month after implantation (session 3)		
OFF state:	35.8±7.0	
sON state:	15.8±4.8	
DBS parameters:	1.6±0.3 V	60–90 µs, 130 Hz, unipolar mode
UPDRS-III – one year after implantation (session 4)		
OFF state:	*not done*	
sON state:	11.4±4.0	
DBS parameters:	2.4±0.5 V	60–90 µs, 130 Hz, unipolar mode

electrode position in the subthalamic area	contact 0	contact 3
right hemisphere (mm):		
x:	8.6±1.2	10.5±1.1
y:	−2.1±1.5	1.7±1.4
z:	−5.3±1.0	−0.1±1.0
left hemisphere (mm):		
x:	−8.2±1.3	−10.4±1.1
y:	−3.5±1.2	0.3±1.5
z:	−5.0±1.1	0.2±1.0

Mean ± SD and variance range is reported for each parameter. MMSE – Mini Mental State Examination. The UPDRS-III was assessed in OFF condition (medication OFF in session 1; medication OFF and STN DBS OFF in sessions 2 and 3) and in mON condition (after administration of 250 mg of levodopa/carbidopa) in session 1 and in sON condition (medication OFF and bilateral STN DBS ON) in sessions 2, 3, 4. DBS parameters – mean amplitude, variance in pulse duration, frequency and mode of stimulation in both hemispheres. Medtronic electrode (type 3389) positions were measured in native space according to methodology [37] on T1-MRI obtained one year after surgery: The x-coordinate of each contact 0 and 3 was measured from the wall of the third ventricle (+ towards right; − towards left), whereas the y-coordinate (+ towards anterior; − towards posterior) and z-coordinate (+ towards vertex; − towards brainstem) were measured from the mid-commissural point.

### Design of the study and clinical assessment

During the study, the patients were examined in three sessions with the UPDRS-III: (session 1) 20± (SD)16 days before implantation, (session 2) on post-implantation day 1 or 3 and (session 3) one month after implantation, each time in the medication OFF and DBS OFF conditions. Four days before the first session, dopamine agonists were substituted by equivalent doses of levodopa. Other anti-PD medication (selegiline, amantadine, anticholinergics) was suspended. In each session, the UPDRS-III was assessed after at least 12 h withdrawal of levodopa. fMRI was assessed during the first two sessions.

All patients were assessed by the UPDRS-III in the ON state as well. Before surgery, they were examined after the administration of 250 mg of levodopa/carbidopa (session 1) and after surgery, they were tested on bilateral STN DBS with stimulation parameters achieving best possible motor improvement. While an external stimulator (Dual Screen 3628, Medtronic, Minneapolis, MN) working in bipolar mode was used in early postoperative phase (session 2), treatment by internal neurostimulator (Kinetra, model 7428, Medtronic, Minneapolis, MN) using unipolar mode of stimulation was initiated one month after surgery (session 3). To evaluate the long-term effects of neurostimulation, all patients were further examined one year after implantation (session 4) in STN DBS ON state with antiparkinsonian medication withdrawn. Stimulation parameters are given in Table 1.

For purposes of the study, scores derived from the UPDRS-III were used to assess rigidity (sum of item 22), akinesia (sum of items 19, 23–26, 31), tremor (sum of items 20, 21) and axial score (sum of items 18, 27–30), which comprised involvement of speech, standing, stability and gait disturbances.

### Surgical procedure and electrode positions

Implantation of the DBS system was performed separately in two steps: (i) stereotactic insertion of the permanent quadripolar electrode into STN bilaterally and (ii) implantation of connection leads and neurostimulator to subclavial region. Leksell frame and SurgiPlan software system (Elekta, Stockholm, Sweden) were employed in stereotactic procedure. Pre-surgical planning was based on MRI with direct visualization of the target. Central trajectory was intentionally focused on the STN center near the anterior part of the red nucleus. The first surgery was performed under local anesthesia and the STN neuronal activity was mapped by parallel insertion of five tungsten microelectrodes spaced 2-mm apart in a “Ben-gun” configuration to select sites for the macroelectrode intraoperative stimulation [35], [36]. The accuracy of planning and surgery was very high as documented by the number of microelectrodes hitting the STN (median 3.5, variance 2–5) in length of 5.4±1.0 mm. The number of tracks used for macroelectrode insertion ranged from 1 to 3 (median 1) and the macroelectrode in track (66% in the central one) eliciting the best clinical outcome was subsequently replaced by a permanent electrode (type 3389). Immediately after the procedure, the position of each permanent electrode was verified by two orthogonal X-ray images co-registered with presurgical MRI plan. No dislocation higher than 1-mm was found in any patient.

Half of the patient group (N = 6) was randomly assigned to have session 2 carried out one day after the implantation, the other half (N = 6) three days after the implantation. On the following day, a neurostimulator was implanted in the subclavian region under general anesthesia. Chronic STN DBS was initiated at the end of the third session which was performed one month after the implantation.

The final positions of permanent electrodes were assessed one year after surgery on T1-weighted images following the previously published approach [37]. Briefly, coordinates of the distal contact “0” and proximal contact “3” were measured manually in native space with regard to line connecting anterior and posterior commissures. Mean positions of contacts are shown in Table 1 and individual coordinates in Table S1. Each of the two contacts was located in a space occupying maximum of 4.0–5.3 mm in each direction which corresponds well to the size and position of the subthalamic area.

### MRI protocols and safety issues

A 1.5-T Siemens Symphony scanner (Siemens, Erlangen, Germany) was used for MRI acquisition. A gradient-echo EPI sequence was used (*TR* = 1000 ms, *TE* = 54 ms) with an in-plane resolution of 3×3 mm^2^, slice thickness of 3-mm and inter-slice gap of 1-mm. A slab of ten oblique slices was acquired, oriented along the central sulcus and covering the rollandic cortex, basal ganglia and thalamus in a region between the anterior border of caudate nuclei and the posterior border of the red nuclei. In total, 500 dynamic scans were acquired during each task. In sessions 2 and 4, axial *T1*-weighted magnetization prepared rapid acquisition gradient echo (MP-RAGE, *TR* = 2140 ms, *TE* = 3.93 ms, 1.65-mm thickness) and *T2*-weighted (turbo-spin echo, *TR* = 5520 ms, *TE* = 86 ms, 4-mm thickness) images were acquired for morphological imaging before and after implantation.

The MRI was performed according to previously defined technical precautions considering the potential hazard in patients with intracerebral electrodes [24], [38]. For thermal risk estimation, temperature changes were measured for each MRI acquisition sequence separately. We used MRI-compatible fluorooptic thermometry [24], [26], [32] (Luxtron Corporation, Santa Clara, CA) in a pig brain phantom, into which four temperature sensors were inserted in the vicinity of the electrode. With the *T1*-weighted sequence, the estimated specific absorption rate (SAR) in the head region was 0.17 W/kg leading to maximal warming by 0.30°C, the *T2*-weighted sequence (SAR = 0.98 W/kg) was associated with a 1.1°C temperature increase and the gradient echo-plannar sequence (SAR = 0.05 W/kg) used for fMRI caused an increase of 0.09°C . To avoid a heating hazard it was necessary to prevent the creation of a loop between the contacts of the implanted electrode and the non-insulated connectors of the extension leads. For that purpose, the externalized leads connectors were properly insulated to avoid direct contact with the patient's skin, then fixed along the z-axis and placed near the center of the MR scanner bore.

### Tapping task and fMRI data analysis

During each of the two fMRI sessions, the patients performed a simple tapping task for each hand separately while lying supine with both hands in a resting position. As performance during motor tasks may affect the fMRI results [39], [40], [41], [42] and frequency of tapping varies in PD [43], each “tap” was individually triggered to ensure low individual variability in motor performance. Each patient was instructed to make a single “tap” consisting in touch of the thumb and index finger of the same hand whenever the “go” signal (yellow square) appeared on the screen. During the task, twenty-five motion and resting periods, each lasting 10 s, were interspersed in an alternating fashion. During each motion period, the “go” signal was displayed ten times with an inter-stimulus interval of 1 s so that 250 movements were performed during the task. Since a microlesion impact on visual functions wasn't expected, an effect of visual stimulation alone was not considered in the experiment. The finger movements were recorded using MRI-compatible gloves (5DT Inc, Irvine, CA) and monitored with a video-camera.

For each patient, fMRI experiments were analyzed separately using SPM8 rev. 4010 (Wellcome Trust Centre for Neuroimaging, UCL, London, UK) and Matlab 7.7 (The MathWorks, Inc, Natick, MA). In the pre-processing phase, the data was realigned to correct for motion artifacts [44], normalized using the unified segmentation approach [45] in standardized stereotactic space (Montreal Neurological Institute – MNI) and smoothed using a Gaussian smoothing kernel of 8 mm full width at half maximum. The electrode caused a strong susceptibility artifact which attenuated the BOLD signal along the entire trajectory including the subthalamic area. These regions were automatically masked-out similar to other non-brain regions by an algorithm implemented in the SPM8. The intra-individual statistical analysis was based on a least-squares estimation using the general linear model and the canonical hemodynamic response function [46], [47]. The design matrix was encoded with the 25 tapping and 25 resting blocks in order to investigate a potential difference between task and baseline, i.e. a potential increase of brain activity during the task compared with rest. Contrast images (encoding task-baseline differences) were computed for all subjects and sessions independently. Those contrast images were used in group analyses using a factorial design with two factors of the moving hand (left/right) and electrode implantation (before/after). The design matrix was generated using 4 columns in order to encode all 4 categories (before-left, before-right, after-left, after-right). The design matrix had 48 rows using 4 contrast images per subject.

The final statistics were based on the contrast *before vs. after implantation* (and vice versa) and displayed using a threshold of *p*<0.001. To suppress false positive results, the family-wise error (FWE) rate was controlled using a corrected threshold of *p*<0.05 at the cluster-level. To control for differences with the UPDRS-III across patients, those values in the OFF state obtained during sessions 1 and 2 were added to the design matrix as an additional column. Using this column as covariate of no interest, differences between the OFF state UPDRS-III scores across patients were taken into account. Analyses were also computed using covariates with the scores of cortical and subcortical oedema in the left and right hemispheres.

The factorial analysis was additionally performed using covariates describing the UPDRS-III score and the cortical and subcortical oedema in the left and right hemispheres. To test the effect of rigidity, tremor, akinesia, and axial scores, separate analyses were done. For regions showing significant results, values were extracted from the 48 contrast images used in the group analyses. A potential correlation between those contrast values and associated UPDRS-III scores was investigated. Further statistical analyses were made with the SPSS 14.0.1 software (SPSS Inc, Chicago, IL). For parameters not following normal distribution, the Mann-Whitney test and Spearman rank correlation were applied. For parameters following normal distribution, analysis of variance with repetition and Pearson correlation analysis were used.

### Oedema assessment

**Figure 1:**
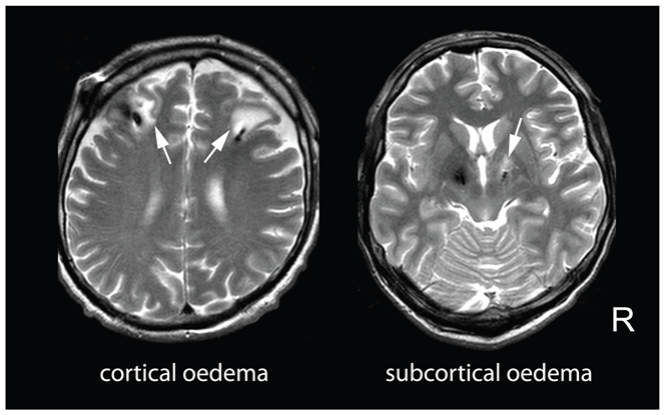
Native *T2*-weighted images of collateral oedema surrounding implanted electrode 3 days after surgery in PD patients. Left – Example of bilateral oedema (score 2) involving frontal cortical regions in patient 4. Right – subcortical oedema (score 2) around contacts of the right electrode involving subthalamus and globus pallidus in patient 3. While the susceptibility artifacts from the electrodes are hypointense, the oedema appears hyperintense (white arrows).

Collateral oedema around implanted electrodes was analyzed by a blinded rater in each patient individually and in each hemisphere separately using the post-operative *T2*-weighted images from session 2, on which oedema appeared as a hyperintense area (Figure 1). To estimate its volume, we developed two semi-quantitative scales for separate assessment of oedema in cortical (affecting the white matter just beyond the cortex) and subcortical (affecting deep structures and adjacent white matter near the electrode) regions: the cortical oedema scale (0 – no oedema; 1 – mild focal oedema near the electrode within the area of one gyrus; 2 – moderate focal oedema near the electrode involving an area larger than one gyrus; 3 – large focal oedema near the electrode involving an area larger than two gyri); the subcortical oedema scale (0 – no oedema; 1 – mild focal oedema near the electrode involving the thalamus and/or mesencephalon; 2 – moderate focal oedema involving the thalamus and/or mesencephalon and/or part of the basal ganglia; 3 – large colateral oedema involving most of the basal ganglia). The total cortical or subcortical oedema score was calculated as a sum of the scores from both hemispheres.

## Results

### Clinical assessment

**Figure 2:**
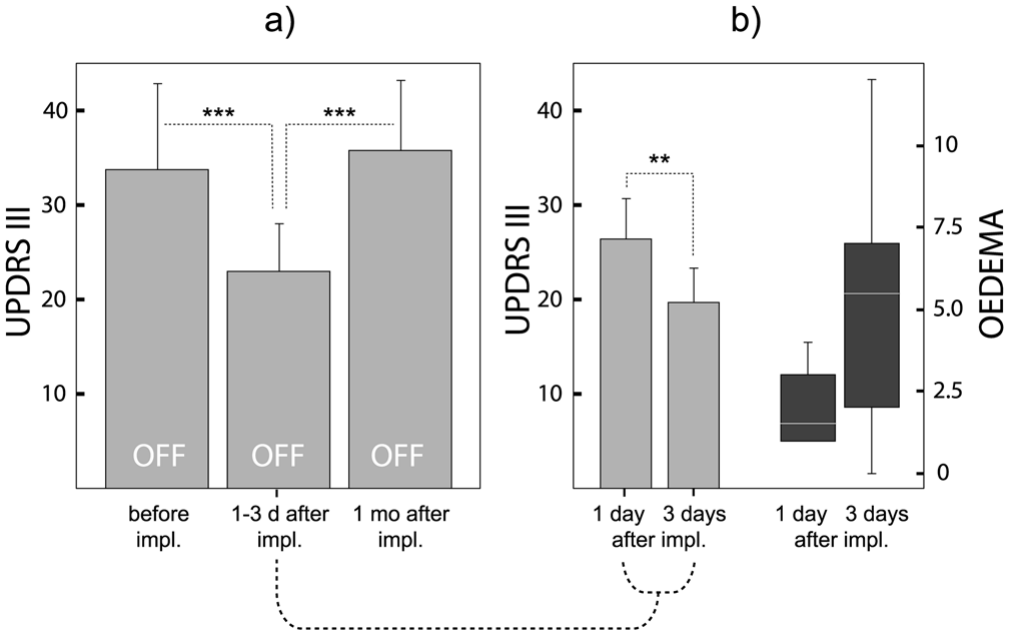
UPDRS-III in OFF conditions and oedema score change in PD patients (N = 12) during the study. a) The UPDRS-III score before implantation (OFF state) in session 1, shortly after implantation of the electrode bilaterally to STN (OFF state) in session 2, and one month after implantation (OFF state) in session 3 examined each time after over-night withdrawal of antiparkinsonian medication and in the absence of DBS. b) PD patients (N = 6) examined 3 days after implantation showed lower UPDRS-III and higher total oedema score than patients (N = 6) examined 1 day after surgery; UPDRS-III – error bars (mean and SD); total oedema score – white line (median), vertical length of the box (interquartile range = IQR), whiskers (smallest score within 1.5 IQR of the lower quartile, and the highest score within 1.5 IQR of the upper quartile); ** (*p*<0.01), *** (*p*<0.001).

The average OFF state UPDRS-III score changed significantly throughout the study (repeated measures ANOVA, *F* = 29, *p*<0.0001). A post hoc analysis revealed a significant UPDRS-III drop from the preoperative score of 33.8± (SD)8.7 in session 1 to 23.3±4.8 in session 2 (*F* = 28, *p*<0.001) within three days after the implantation (Figure 2a). One month after surgery, the OFF state UPDRS-III score significantly increased to 35.8± (SD)7.0 (*F* = 54, *p*<0.0001) nearly returning to the pre-implantation value so that no difference between sessions 3 and 1 could be detected (*F* = 0.9, *p* = 0.4). Rigidity, akinesia and axial scores (but not the tremor score) dropped within three days of the surgery and resumed to their previous value one month later similar to changes in the UPDRS-III score (*F* = 55, *p*<0.0001). In the six patients examined one day after surgery, the OFF state UPDRS-III decreased by 24% (*p*<0.05) and in the other six patients examined three days after surgery decreased by 33% (*p*<0.01). While pre-operatively, the OFF UPDRS-III score between these groups did not differ (*p* = 0.2), post-operatively, the score was significantly lower in the group of patients examined three days after implantation (*p*<0.01)(Figure 2b).

**Figure 3:**
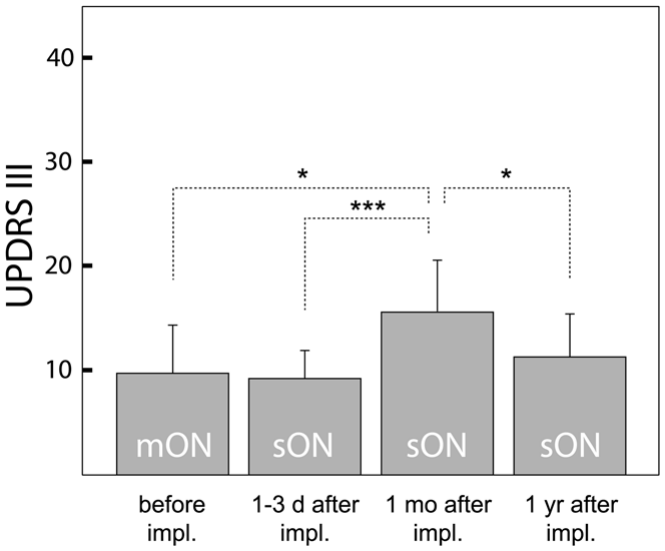
UPDRS-III in ON condition in PD patients (N = 12) during the study. The UPDRS-III score before implantation (mON) in session 1 after administration of 250 mg levodopa/carbidopa; in session 2 within 1–3 days after implantation of the electrode bilaterally to STN; in session 3 one month after implantation; and in session 4 one year after implantation examined each time with STN DBS ON (sON state) always with antiparkinsonian medication withdrawn; UPDRS-III – error bars (mean and SD); * (*p*<0.05), *** (*p*<0.001).

The UPDRS-III decreased with STN DBS switched ON compared to OFF state by 60% in session 2 and by 55% in session 3 which is comparable to 71% preoperative decrease with levodopa in session 1. However, the UPDRS-III differed among ON conditions in all four sessions (repeated measures ANOVA, *F* = 8.8, *p*<0.01)(Figure 3). The post hoc analysis revealed that the ON state UPDRS-III significantly increased one month after surgery compared to preoperative session 1 (*F* = 7.2, *p*<0.05), to early postoperative session 2 (*F* = 23, *p*<0.001) and in comparison with the postoperative session 4 (*F* = 6.0, *p*<0.05). In addition, the preoperative ON state UPDRS-III after levodopa intake did not differ from postoperative conditions with neurostimulation switched ON in session 2 (*F* = 0.1, *p* = 0.7) and in session 4 (*F* = 0.8, *p* = 0.4).

In any session and in any OFF/ON conditions, the UPDRS-III did not correlate with the number tracks made by microelectrodes hitting the STN or by macroelectrodes used for intraoperative clinical testing.

### Oedema assessment

The median score of postoperative cortical oedema around the implanted electrodes in session 2 was 1.5 (0–6), the subcortical oedema median being 1.0 (variance 0–6). The majority of patients (N = 7) showed signs of oedema at both cortical and subcortical levels. One patient had no signs of oedema at all, three had developed oedema subcortically, and one patient near the cortex. In 10 out of 11 patients, the oedema occurred asymmetrically affecting predominantly one hemisphere. In comparison to preoperative OFF state UPDRS-III, the subcortical oedema score correlated with the OFF state UPDRS-III decrease examined before and within three days of the implantation (*rho* = −0.58, p<0.05) but not with change in the OFF state UPDRS-III one month later. In addition, the oedema total score tended to grow (*p*<0.1) during the first three days after surgery from 1.5 (1–4) to 5.5 (0–12)(Figure 2b). No correlations of the oedema score with number microelectrode tracks in the STN or with number of macroelectrodes were detected. In addition, no signs of collateral oedema were observed on the *T2*-weighted images one year after surgery.

### Tapping test in fMRI

**Figure 4:**
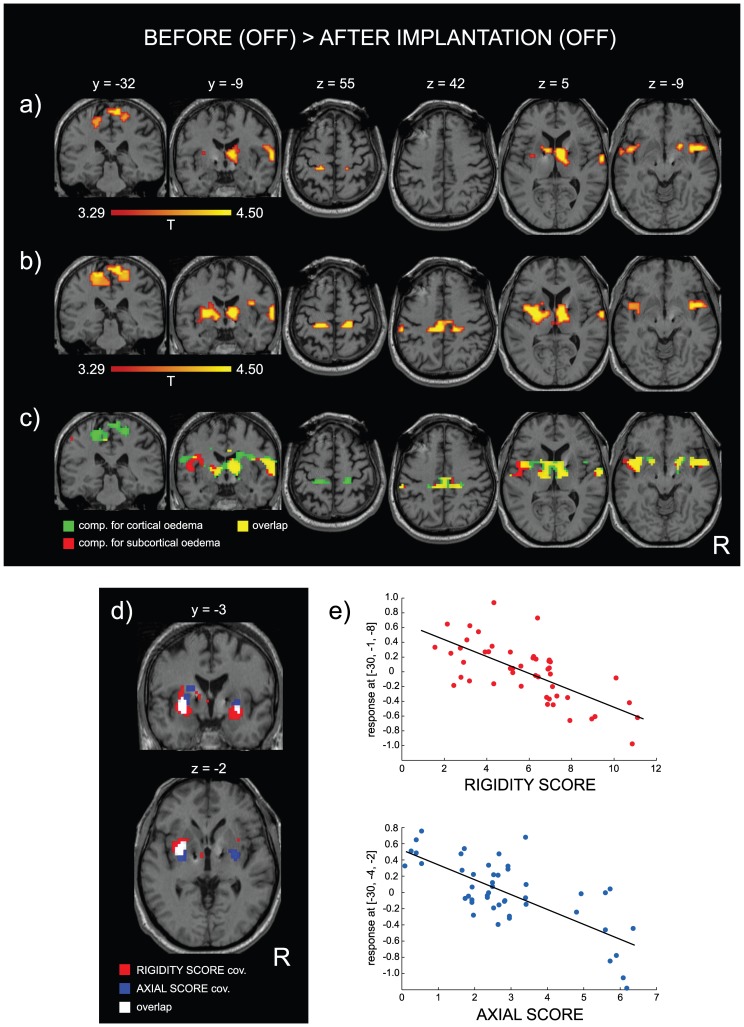
fMRI group analysis of the tapping test performed by PD patients (N = 12) before and after implantation of the electrodes bilaterally to the STN in the absence of DBS. Color-coded clusters show areas with BOLD signal decrease (*p*<0.001 uncorrected) after implantation (OFF state) as compared to before implantation (OFF state). Color-coding shows *T* values greater than 3.29 which corresponds to *p*<0.001. The location of the coronal and axial slices is shown using the y and z component within the MNI space. Contrast *before vs. after implantation*: a) without any covariate; b) with the UPDRS-III as a covariate showing general improvement of contrast in nearly all regions; c) with the cortical and subcortical oedema as covariates enhancing contrast in slightly different regions: the cortical oedema covariate influenced mainly motor cortices (green), the subcortical oedema covariate improved contrast especially in the insula (red). Overlap between both covariates is shown as well (yellow). d), e) Effect of normalized rigidity score (red) and axial score (blue) covariates expressed by significant inverse correlation with the BOLD signal in the putamen and globus pallidus in PD patients regardless to effect of implantation (d) and shown by linear regression (e) in two voxels with local maxima (*x*, *y*, *z* in the MNI space) at the left putamen. Response on the y-axis represents the size of the effect of interest expressed in relative values from comparison of the motor task vs. rest. Each patient is represented by four dots because of the four tasks performed (left/right hands and before/after implantation). The overlap is shown in white (d).

The fMRI was completed by each patient with no dystonia or dyskinesia manifested during the task. In both sessions, an expected activation of the contralateral primary sensorimotor cortex (SM1), supplementary motor area (SMA) and basal ganglia had been observed during the tapping test. However, a comparison based on contrast *before vs. after implantation*, yielded a significant BOLD signal difference in several cortical and subcortical areas (Figure 4a, Table 2). At the cortical level, movement-related activation was found preoperatively higher in the bilateral primary sensorimotor cortex (SM1), middle part of insula, and in the rollandic operculum involving the inferior postcentral and superior temporal gyri than postoperatively. At the subcortical level, the BOLD signal was preoperatively higher in the right anterior, dorsal and lateral thalamus.

**Table 2:** fMRI group analysis of the tapping test performed by PD patients (N = 12) before and after implantation of the electrodes bilaterally to the STN in absence of DBS.

	UPDRS-III cov.							oedema cov.		
	x	y	z	k	T†	*p* †		T#	*p* #	
**a) before vs. after-implantation** (BOLD decrease):										
precentral g. (SM1)										
right	21	−28	58	113	4.69	10^−5^	**	4.51	10^−5^	***
left	−21	−31	52	410‡	4.63	10^−5^	***	4.62	10^−5^	***
middle cingulate and SMA	3	−25	40	410‡	4.40	10^−4^	***	3.56	10^−4^	***
rollandic operculum										
right (OP4)	66	−7	10	44	4.30	10^−4^	*	5.47	10^−6^	***
left (OP1)	−60	−16	10	11	4.21	10^−4^		4.26	10^−4^	***
thalamus										
right (anterior/dorsal)	9	−3	6	410‡	4.24	10^−4^	***	4.72	10^−5^	***
right (lateral)	15	−13	4	410‡	4.49	10^−4^	***	5.33	10^−5^	***
left (anterior/dorsal)	−12	−7	1	326	3.67	10^−3^	***	4.70	10^−5^	***
left (lateral)	−13#	−11#	1#		3.57	10^−3^		4.13	10^−4^	***
putamen and globus pallidus										
right	18#	6#	−6#		2.81	10^−2^		4.46	10^−4^	***
left	−27	−4	10	326	4.11	10^−4^	*	3.66	10^−4^	***
insula										
right	48	8	−8	81	4.32	10^−4^	**	5.19	10^−5^	***
left	−42	5	−5	410‡	4.33	10^−4^	***	3.66	10^−4^	***
**b) rigidity score** (BOLD decrease):										
putamen and globus pallidus										
right	30	−1	11	96	4.33	10^−4^	**			
left	−30	−1	−8	223	4.88	10^−6^	***			
**c) akinesia score:**										
*none*										
**d) tremor score:**										
*none*										
**e) axial score** (BOLD decrease):										
putamen and globus pallidus										
right	30	−7	−2	40	3.94	10^−4^				
left	−30	−4	−2	112	4.82	10^−5^	**			

a) Contrast showing decrease of the BOLD signal after implantation (OFF state) as compared to situation before implantation (OFF state) with the UPDRS-III or the oedema score as covariates; x, y, z – local maxima of clusters in MNI coordinates derived from the contrast *before vs. after implantation* with the UPDRS-III covariate; k – size of the cluster in voxels;

‡ - regions belonging to the same cluster; T – T-score; *p* – uncorrected level of significance;

* (*p*<0.05),

** (*p*<0.01),

*** (*p*<0.001) – significance with FWE correction at cluster-level;

† – values derived from the contrast *before vs. after implantation* with the UPDRS-III covariate;

# – values derived from the contrast *before vs. after implantation* with the oedema covariate.

b), c), d), e) – Impact of the UPDRS-III sub-scores on size of the BOLD response expressed as inverse effects of rigidity and axial covariates.

Using the UPDRS-III score as a covariate, the contrast *before vs. after implantation* became even more pronounced (Figure 4b). The preoperative BOLD signal increase was observed to be significantly higher especially in the supplementary motor area (SMA) and bilaterally in the SM1, insula, thalamus, putamen and in the globus pallidus in comparison with the examination after surgery. When the oedema score was considered, the contrast *before vs. after implantation* became bilaterally stronger in the basal ganglia, SMA and insula (Figure 4c). To search for a possible postoperative BOLD signal increase, all analyses were also performed using the contrast *after vs. before implantation* with no significant results found.

In addition, there were significant effects of rigidity and axial scores in the fMRI group results. Their effects were even stronger than the original UPDRS-III covariate. The BOLD signal intensity correlated inversely with the normalized rigidity score (*r* = −0.69, *p*<0.0001) and with the normalized axial score (*r* = −0.65, *p*<0.0001) in the putamen and globus pallidus bilaterally (Figure 4d,e). The effect of the rigidity score involved a larger volume of these structures and was located more antero-inferiorly than the axial score effect.

## Discussion

### Impact of microlesion on motor performance

Our PD patients showed significant clinical improvement manifested by a 24% reduction in the OFF state UPDRS-III in the subgroup of patients examined the first day after the electrodes were implanted and even a 33% reduction in patients examined on the third postoperative day (Figure 2b). This improvement was seen especially in rigidity and akinesia but also in axial symptoms such as gait, postural stability and speech and disappeared one month after surgery. Hence, we corroborate previous clinical effects of unintended microlesion as a result of the electrode insertion in the target nucleus [2], [3], [4], [7], [8], [10].

The effect of STN DBS ON vs. OFF state with motor improvement by 60–55% in sessions 2–3 is comparable to previous reports [48], [49] and indicates correct placement of the permanent electrodes. However, the ON state motor performance varied throughout the study suggesting that microlesion affected the responsiveness to the neurostimulation as well. Contrary to OFF state after withdrawal of antiparkinsonian medication, i.e. to condition in which the effect of microlesion is easily recognized, it is difficult to draw any conclusions based on comparisons among sessions in the ON state as they may reflect different way of treatments (levodopa challenge in session 1, different amplitude of STN DBS with bipolar mode in sessions 2 and 3 or unipolar mode in session 4). Assuming patients reached their best ON, i.e. the parameters of treatment were optimal in each session, making such a comparison is feasible (Figure 3). We observed that the effects of STN DBS alone one month after surgery did not reach the efficacy of levodopa before surgery while the effects of STN DBS together with microlesion in session 2 were nearly equal to preoperative effects of levodopa. This may support the hypothesis of additional effect of microlesion to the effects of STN DBS as both seem to be cumulated. The positive result is that after one year, the STN DBS became more efficacious compared to situation one month after surgery when chronic neurostimulation was initiated. Thus the clinical signs of microlesion effect obviously had no impact on future long-term responsiveness to STN DBS.

### Mechanisms of action

The acute microlesion effect may act by several mechanisms. First, damage and subsequent loss of part of the neuronal population and glial cells [50], i.e., micro-subthalamotomy, to which the patients respond by motor improvement similar to conventional subthalamotomy [5], [51]. Weeks and months after insertion of the DBS electrode, unknown compensatory mechanisms are probably induced, which gradually suppress the microlesion effect and finally worsen the UPDRS score [7]. We observed this in our study as well. One month after surgery, the OFF state UPDRS-III score had returned to nearly the preoperative level (Figure 2a) suggesting complete abatement of the microlesion effect. To the contrary, Mann at al. observed OFF state clinical improvement several months after implantation implying that the microsubthalamotomy can persist even for a long period of time [7] or it could just be a consequence of a neurostimulation after-effect due to DBS being switched-off for an insufficient period of time. On the other hand, using more microelectrodes for exploration during surgery resulted in greater tissue damage and in even more pronounced OFF state improvement [10]. We were unable to confirm this observation since the number of tracks through the STN made by any from the five exploratory microelectrodes did not correlate with postoperative improvement in either OFF or ON conditions compared to the preoperative state. Also the number of stimulation macroelectrode insertions which may lead to greater tissue contusion than microelectrodes was unrelated to the postoperative OFF or ON state motor improvement.

Another mechanism of action may rely on release of various neurostransmitters [52] due to penetration of the electrode into subthalamic area. It is especially gama-aminobutyric acid (GABA) as main inhibitory substance on the STN input [53] and glutamate as main excitatory mediator on its output [52], [54]. Uncontrolled leakage of neurotrasmitters may severely influence the function of undamaged neurons and thus significantly contribute to effects of microlesion in the acute phase.

Finally, probably more significant mechanism of the microlesion effect in its acute phase should be sought in the collateral oedema (Figure 1) which was found in most of our implanted patients (92%). This was suggested by a postoperative decrease of the OFF state UPDRS-III being more pronounced in patients examined after three days than in patients seen the first day after implantation and by its final increase to its preoperative value one month later (Figure 2b). Using simple semiquantitative scales for rating the brain oedema on *T2*–weighted images we were able to document its impact on motor performance. As expected, the UPDRS-III decline in the early postoperative phase correlated inversely with the grade of subcortical but not cortical oedema. The histopathological picture of collateral oedema near the electrode [33] suggests similar mechanisms similar to common traumatic brain oedema [55]. Damaged neurons show axonal swelling with subsequent dieback of distal segment accompanied by proximal swelling developing within 48 hours after injury [56]. Intracellular (cytotoxic) swelling of astrocytes [57] facilitated by aquaporins [58], membrane channels regulating water permeability, predominates in acute stage. Subsequently, changes in the transmembrane ionic gradients develop leading to depolarization of cells [59]. Finally, extracelluar (vasogenic) swelling develops due to blood-brain barrier dysfunction accompanied by local production of numerous inflammatory mediators [55]. Contrary to permanent neuronal damage responsible for long-term effects of microlesion, these changes are only temporary suggesting why clinical consequences of microlesion are maximally expressed within the first days after implantation.

Collateral brain oedema in implanted patients has already been observed around deep [3], [34], [60] as well as strip electrodes with a relatively low incidence of 1–2% [61], [62]. The frequent detection of oedema in our study may have to do with our specific aim to search for even its minimal signs early after surgery. These were often manifested just by suppression of a previously hypointense susceptible artifact resulting from the metallic electrode. This phenomenon was very visible mainly during inter-hemispheric comparison when only one of the electrodes was surrounded by oedema (Figure 1). However, there is no clear explanation for the inter-hemispheric asymmetry or variability of the oedema score in individual patients. It could not have been associated with the number of the macroelectrode trajectories nor with other known aspects of the surgery.

### Relative cortical and subcortical hypoactivation in the OFF state

Small spontaneous thalamic lesions after stroke have shown to significantly affect the cortical functions [63]. Our group fMRI results showed that even the transitory STN microlesion may have a significant impact on the cortical pattern in PD patients when examined without medication and without neurostimulation. The movement-related BOLD increase in the motor network occurred in both sessions, but the activation was relatively less pronounced after surgery than when compared to the preoperative condition giving a picture of postoperative hypoactivation in a number of cortical and subcortical motor areas. We regard the generally insufficient movement-related activation shortly after surgery as a substantial finding in our study (Figure 4). The less pronounced activation occurred not only relatively close to the electrodes, such as in the anterior thalamus, putamen and globus pallidus, but also in distant regions such as the primary sensorimotor cortex (SM1), secondary sensory cortex (SII), supplementary motor area (SMA) and insula. This pre-/postoperative contrast was further enhanced when the UPDRS-III covariate was used to compensate for the inter- and intra-individual variability of motor impairment (Table 2, Figure 4a, b) suggesting that varying motor involvement was not responsible for this insufficiently low movement-related activation after surgery.

It should be mentioned that this finding was not an artifact arising from possible BOLD signal attenuation due to the physical features of oedema because all the relatively less active areas were well outside the regions affected by oedema. In addition, with the group analysis compensated for the cortical and subcortical oedema score, the pre−/−postoperative contrast was further enhanced (Table 2, Figure 3c) implying that the oedema alone cannot explain all of our fMRI results. It is probably the neuronal and glial damage in the electrode trajectory which plays a major role in microlesion effect on motor system functioning a few days after surgery.

As there are no previous fMRI studies with electrode insertion we may compare our results only with PET studies describing reduction in the basal ganglia activity as a consequence of microlesion [12] or even subthalamotomy [13], [14]. However, these studies analyzed PD patients in a resting state while we refer to microlesion-related subcortical and cortical hypoactivation during movements in comparison to rest. fMRI studies analyzing movements in PD in the OFF medication state usually found decreased [64], [65], [66], [67], [68], [69] or unchanged [15], [16], [39], [70], [71], [72] activity in basal ganglia with cortical motor regions being variably involved in comparison to control subjects perhaps due to various stages of PD and different aspects of movement tasks. As the control group was missing in our study we can only compare our results with fMRI studies focusing on effects of treatment like DBS or levodopa. In contrast to the microlesion effect, the STN DBS-treated patients exhibited a movement-related increase in thalamic and basal ganglia activation [73], [74] whereas SMA is more often described as increased [73], [75], [76] than decreased [74]. In addition, the SM1 was relatively less active during movements with STN DBS switched on [30], [74], [77], [78] similar to our PD patients with microlesion. Administration of levodopa seems to normalize hypoactivation in basal ganglia [79], [80] or SMA [15], [18] accompanied by an increase of activity in previously hypoactive SM1 [15] or decrease of activity in previously hyperactive SM1 [18] cortex. Other authors described more focalized [16] or unchanged activity [79], [80], [81] in the SM1 in response to levodopa challenge. The mechanisms of microlesion thus seem to differ from known effects of levodopa. We came to this conclusion after our recent fMRI study in PD patients studied in both medication OFF/ON conditions using the same task [82]. Surprisingly, the motor improvement after levodopa was accompanied by an increased activation in basal ganglia, while in the present study, motor improvement after electrode insertion was accompanied by hypoactivation in these structures.

The interpretation of our results in context of basal ganglia models [83], [84], [85] is difficult as the BOLD fMRI rely on local perfusion and oxygenation [86] reflecting neuronal activity only indirectly and irrespective to activation and inhibition, i.e. terms these models usually refer to. The STN is part of the indirect striato-pallido-thalamo-cortical pathway which is believed to be hyperactive in the OFF state. One may assume that the microlesion will suppress abnormally high STN neuronal activity [87] and subsequently down-regulate the excitatory output of indirect pathway projecting to the globus pallidus [52]. Unfortunately, the STN activity cannot be detected due to the electrode artifact but we see consequence of the STN microlesion expressed indeed as pallidal hypoactivation. In contrary, administration of levodopa is associated with basal ganglia hyperactivation [79], [80], [82] perhaps because of up-regulation in the direct pathway. An explanation of why we saw deactivation in the SM1 and SMA with microlesion is difficult despite their tight effective connection with STN [88] via hyper-direct pathway [89], [90]. We speculate that the SM1 and SMA hypoactivation might be attributed to neuronal dysfunction in cortex caused by antidromic propagation of dieback, i.e. phenomenon already described on axons in visual system after local traumatic injury [56]. Relatively low activation of the insula, which we postoperatively observed in both hemispheres, is usually reported to be increased during active STN DBS [30], [32]. The insula is known to be involved in the mechanisms of subjective interoception and nociception [91]. Hence we cannot rule out the possibility that a change in its activity was related to subjective perception of altered rigidity and akinesia.

### UPDRS-III on fMRI results

A specific BOLD signal behavior in the putamen and globus pallidus, regardless of the pre/postoperative state, is a separate result. Lower rigidity and axial scores were associated with basal ganglia hyperactivation (Table 2, Figure 4d, e) during movements which may account for the pathophysiological nature of these symptoms in PD. This seems to be in contradiction with our observation of relatively deactivated basal ganglia due to microlesion (Figure 4a) because patients were clinically better few days after surgery. An explanation is in how the UPDRS-III was used in the analyses. For the contrast *before vs. after* implantation shown on Figure 4b the UPDRS-III was used as a covariate (nuisance vector), which means that fMRI data heterogeneity reflected in varying UPDRS-III had been mathematically “removed”. Indeed, the general postoperative hypoactivation became more distinct after this removal (compare Figure 4a and b) suggesting that the UPDRS-III had no relation to the microlesion effect on the fMRI neither like the oedema score (Figure 4c) and that the microlesion affected fMRI results of all patients in similar manner. Our observation of negative correlation of basal ganglia activity with rigidity and axial scores is based on opposite use of the UPDRS-III in the group statistics. Instead of removal, the UPDRS-III was considered as effect of interest with the microlesion factor suppressed. To summarize both results, the motor improvement was associated with increasing basal ganglia activity during motor task but this activity was superimposed on the lower baseline after surgery due to microlesion effect.

Worsening of rigidity and axial symptoms with increasing BOLD signal in the putamen and globus pallidus during motor task is in line with previous observations. Using the grip force task, Prodoehl et al. described a negative correlation between UPDRS-III with the activation of basal ganglia in the OFF state in levodopa naïve patients [66]. Similarly, there was a negative correlation of the UPDRS-III with activation of putamen in OFF condition during uni/bimanual coordination task [69], with effective connectivity of basal ganglia to cortical motor regions and cerebellum during the tapping task [92] or with the density of presynaptic striatal dopamine transporters in the OFF condition at rest [93]. The novel finding is that the negative correlation between motor symptoms intensity and movement-related basal ganglia activity was present not only before but also after surgery, because the STN microlesion had no obvious impact on this relationship. Besides that, the BOLD signal relation to rigidity and axial symptoms of our patients showed a partially different spatial distribution in the basal ganglia supporting a specific somatotopic organization of those structures [94], [95], [96].

### Technical limitations

The use of postoperative MRI with the presence of externalized implanted electrodes was free from any complications. We corroborate previous problem-free use of MRI in implanted patients for structural [21], [22], [23] as well as functional [28], [30], [32] imaging. According to our phantom experiments, fMRI carries a lower risk of thermal damage than conventional *T1*– or *T2*–weighted imaging which is also in agreement with previous observations [26], [97]. What we regard as important is compliance with several recommendations [24], [25], [98] involving measurement of temperature changes after each modification of the acquisition sequence as well as proper insulation and specific placement of the externalized leads.

There are several limitations to our study. We cannot fully exclude that the STN was somehow stimulated by the permanent electrode due to currents induced by RF pulses during fMRI. Since their frequency was in the radiofrequency range and that the loop between contacts and leads was open we assume that the neuronal effect of induced currents is rather small or negligible. The neuronal membrane is able to react on stimulation frequencies up to 10 kHz which is far beyond the frequencies occurring in MRI [99]. On the other hand, the radiofrequency field transmitted by the implanted electrode is always associated with local temperature increase in the vicinity of contacts, which may be another mechanism of neuronal stimulation. Warming causes firing rate increase in termosensitive and not in temperature insensitive neurons of hypothalamus (structure which also detects brain temperature) in 32–38°C temperature range [100]. Therefore it seems unlikely that warming of the electrode was responsible for the fMRI results since the temperature change measured on phantom at the end of the fMRI experiment was very low (0.09°C) neglecting further cooling by blood circulation.

## Conclusions

Our results corroborate previous observations that the microlesion produced by an electrode penetrating the STN leads to motor improvement in PD patients even with medication suspended and DBS switched off. Microlesion is very common, and the collateral oedema appears to be co-responsible for its effect in the early postoperative phase. In addition, the microlesion had a significant effect on fMRI pattern elicited by simple finger movements manifested by generally lower activation in the cerebral motor network, which partially differed from known effects of DBS or levodopa. The presence of microlesion does not automatically mean worse outcome with STN DBS in future and from perspective of long-term efficacy seems have no predictive value. Moreover, the clinical symptoms of the microlesion disappeared within one month after implantation, documenting that its effect is not permanent and advocating for delaying an initiation of chronic STN DBS for at least a few weeks after surgery.

## Supporting Information

Table S1 **Individual description of the PD patient's group (N = 12).** ID – patient's identification number; age – age of patient at surgery in years; G – gender: male (M); DD – duration of the PD in years; LD – duration of the levodopa treatment in years; MC – duration of motor complications in years; UPDRS-III in sessions 1–4: First number refers to OFF condition (medication OFF in session 1; medication OFF and STN DBS OFF in sessions 2 and 3), second number refers to mON condition (after administration of 250 mg of levodopa/carbidopa) in session 1 and to sON condition (medication OFF and bilateral STN DBS ON) in sessions 2, 3, 4. ND – not done in session 4; H – right (R) and left (L) hemisphere; MIE – number of microelectrodes out of 5 microelectrodes which reached the STN during the exploration phase of the surgery; Le – length (mm) of the STN measured by the microelectrode with the longest hit; Tr – microelectrode trajectory (c: central, m: medial, l: lateral, a: anterior, p: posterior) finally used for permanent electrode; MAE – number of macroelectrode trajectories used for perioperative clinical testing; SCO – subcortical oedema score; CO – cortical oedema score; coordinates od the permanent electrode (Medtronic, type 3389) contact 0 and contact 3 were measured in native space according to methodology [37] on T1-MRI obtained one year after surgery. The x-coordinate was measured from the wall of the third ventricle (+ towards right; − towards left), whereas the y-coordinate (+ towards anterior; − towards posterior) and z-coordinate (+ towards vertex; − towards brainstem) were measured from the mid-commissural point.
(DOCX)
