# Supplementary material for: The Subthalamic Microlesion Story in Parkinson's Disease: Electrode Insertion-Related Motor Improvement with Relative Cortico-Subcortical Hypoactivation in fMRI
Source: PLoS One. 2012 Nov 7;7(11):e49056. doi: 10.1371/journal.pone.0049056 (PMC3492182; doi:10.1371/journal.pone.0049056)
Supplement: Table S1 — Individual description of the PD patient's group (N = 12). ID – patient's identification number; age – age of patient at surgery in years; G – gender: male (M); DD – duration of the PD in years; LD – duration of the levodopa treatment in years; MC – duration of motor complications in years; UPDRS-III in sessions 1–4: First number refers to OFF condition (medication OFF in session 1; medication OFF and STN DBS OFF in sessions 2 and 3), second number refers to mON condition (after administration of 250 mg of levodopa/carbidopa) in session 1 and to sON condition (medication OFF and bilateral STN DBS ON) in sessions 2, 3, 4. ND – not done in session 4; H – right (R) and left (L) hemisphere; MIE – number of microelectrodes out of 5 microelectrodes which reached the STN during the exploration phase of the surgery; Le – length (mm) of the STN measured by the microelectrode with the longest hit; Tr – microelectrode trajectory (c: central, m: medial, l: lateral, a: anterior, p: posterior) finally used for permanent electrode; MAE – number of macroelectrode trajectories used for perioperative clinical testing; SCO – subcortical oedema score; CO – cortical oedema score; coordinates od the permanent electrode (Medtronic, type 3389) contact 0 and contact 3 were measured in native space according to methodology [37] on T1-MRI obtained one year after surgery. The x-coordinate was measured from the wall of the third ventricle (+ towards right; − towards left), whereas the y-coordinate (+ towards anterior; − towards posterior) and z-coordinate (+ towards vertex; − towards brainstem) were measured from the mid-commissural point. (DOCX) [file pone.0049056.s001.docx]

**Table S1**

**permanent electrode**

**session 1 session 2 session 3 session 4 exploration oedema contact 0 contact 3**

**ID age G DD LD MC OFF/mON OFF/sON OFF/sON OFF/sON H MIE Le Tr MAE SCO CO x y z  x y z**

1 63 M 15 13 12 21/5 25/10 39/23 ND/8 R 4 7.0 c 1 0 0 6.7 -4.6 -4.3 9.4 -1.0 0.7

L 2 4.5 m 1 0 2 -5.5 -4.1 -4.7 -8.3 -0.4 0.5

2 53 M 11 7 3 45/9 32/13 42/19 ND/17 R 5 4.5 c 1 0 0 9.9 -0.8 -4.9 11.7 3.2 0.1

L 5 6.5 p 2 1 0 -8.0 -4.1 -2.6 -10.8 -0.1 2.2

3 46 M 15 12 9 40/13 25/6 38/14 ND/11 R 4 5.0 c 1 2 3 8.5 -3.9 -5.1 10.4 0.2 -0.3

L 4 5.0 c 1 0 0 -10.0 -3.6 -4.8 -11.6 0.8 0.2

4 64 M 14 13 4 31/2 15/6 31/18 ND/8 R 4 5.0 c 1 2 2 6.9 -0.5 -5.1 8.6 2.9 0.1

L 3 6.0 c 2 1 2 -6.1 -5.6 -6.4 -8.7 -1.7 -1.5

5 58 M 11 8 3 26/11 21/7 18/9 ND/5 R 5 5.5 c 2 1 1 9.9 -4.0 -5.4 11.0 -0.5 0.5

L 5 7.0 c 1 0 0 -9.3 -3.8 -6.7 -10.8 -0.5 -0.8

6 49 M 9 5 2 21/9 18/11 33/23 ND/8 R 5 4.0 a 3 0 0 7.8 -0.3 -4.3 9.7 2.7 1

L 3 5.5 m 2 0 0 -7.8 -3.9 -6.4 -9.4 -0.5 -0.8

7 64 M 14 13 5 37/11 23/11 33/12 ND/16 R 3 3.5 a 1 2 0 9.4 -1.6 -6.2 11.1 2.3 -0.7

L 2 4.5 a 2 2 2 -8.8 -1.3 -4.7 -11.4 2.7 0.1

8 53 M 12 10 5 37/11 29/13 38/12 ND/11 R 3 5.0 c 1 1 0 8.1 -2.8 -6.1 10.5 0.8 -1.4

L 5 5.0 c 1 0 0 -9.2 -2.6 -3.8 -11.3 1.5 1.1

9 59 M 9 8 2 26/6 18/8 30/12 ND/13 R 2 4.0 c 1 3 3 9.3 -2.8 -3.2 11.3 1.7 2.2

L 3 6.0 c 1 3 3 -9.8 -1.5 -4.1 -11.3 3.1 1.0

10 45 M 14 6 6 47/21 28/9 45/16 ND/10 R 4 4.5 a 3 1 0 10.8 -0.4 -6.1 12.8 2.8 -0.4

L 2 5.5 c 1 0 0 -7.8 -5.0 -4.6 -10.4 -1.9 1.1

11 64 M 13 8 6 31/10 21/12 39/21 ND/19 R 2 6.5 c 1 1 1 8.4 -2.1 -6.8 10.5 1.8 -1.8

L 4 6.0 c 1 0 1 -8.3 -3.5 -5.4 -11.0 -0.3 -0.5

12 53 M 12 9 3 43/10 24/6 43/10 ND/11 R 3 6.5 m 2 2 1 7.2 -0.8 -6.1 9.5 3.2 -0.7

L 3 7.0 p 1 1 0 -7.3 -2.6 -5.3 -9.7 0.7 -0.5
